# Supplementary material for: Reduced brain entropy in migraine with partial restoration during attacks: A resting-state fMRI study
Source: medRxiv. 2025 Oct 31:2025.10.29.25339059. Preprint. [Version 1] doi: 10.1101/2025.10.29.25339059 (PMC12636680; doi:10.1101/2025.10.29.25339059)
Supplement: Supplement 1 [file media-1.pdf]

Title

Reduced brain entropy in migraine with partial restoration during attacks: A resting-state fMRI study

Authors

Saberi Majid[1], Dajung J Kim[1], Xiao-Su Hu[1], Alexandre F DaSilva[1*]

1- Headache and Orofacial Pain Effort (H.O.P.E.), Biologic and Materials Science & Prosthodontics, University of Michigan School of Dentistry, Ann Arbor, MI, USA

**Corresponding Author:*

adasilva@umich.edu

Region	HC vs EM	HC vs CM	EM vs CM
OC	1.88e-09 (2.16)	5.05e-06 (1.81)	0.57 (-0.35)
rSMG+rSPL	2.52e-03 (0.92)	1.62e-11 (2.81)	1.7e-07 (1.89)
PCu+PCC	7.84e-02 (0.71)	1.53e-07 (2.3)	2.09e-04 (1.59)
mPFC	2.18e-05 (1.61)	3.84e-08 (2.49)	6.37e-02 (0.88)

Table S1. Group-level comparisons of regional mean entropy values. Cells denote adjusted p-values, with t-values in parentheses. Comparisons are shown for healthy controls (HC), episodic migraine (EM), and chronic migraine (CM) across four clusters identified in the main analysis (Figure 1): occipital cortex (OC), right supramarginal gyrus and superior parietal lobule (rSMG+rSPL), precuneus and posterior cingulate cortex (PCu+PCC), and medial prefrontal cortex (mPFC). All clusters survived cluster-level FWE correction.
